# Simulation of a Dual-Band Reconfigurable Metasurface Absorber with Independent Absorption Intensity and Frequency Tuning

**DOI:** 10.3390/ma19122543

**Published:** 2026-06-12

**Authors:** Ting Qin, Yuchen Han, Yujie Gao, Run Mao, Shuang Chen, Jianyun Shi, Junxiong Guo

**Affiliations:** 1 School of Electronic Information and Electrical Engineering, Chengdu University, Chengdu 610106, China; qinting@cdu.edu.cn (T.Q.); 17341873033@163.com (Y.G.); maorun@cdu.edu.cn (R.M.); chenshuang@cdu.edu.cn (S.C.); 2 School of Physics, Central South University, Changsha 410083, China; yuchenhan_2023@163.com; 3 Hardware Research & Development, TD Tech Limited, Chengdu 610041, China

**Keywords:** metasurface, reconfigurable absorber, dual-band, independent control, equivalent circuit model

## Abstract

Metasurface absorbers play a critical role in microwave electromagnetic control, yet conventional designs suffer from fixed performance and strong cross-coupling between tunable parameters, limiting their adaptability in dynamic environments. Here, we propose a dual-band reconfigurable metasurface absorber with independent modulation of absorption intensity and frequency. The absorber adopts a double-layer metallic structure integrated with PIN diodes and varactors, realizing independent regulation of absorption intensity and frequency. In the lower band (4.1–7.7 GHz, S_11_ < −10 dB), the absorption intensity is continuously tunable via the PIN diode bias without frequency shift, while in the upper band (13.4–14.4 GHz), the absorption frequency is continuously tunable via the varactor bias without intensity variation. Quantitative cross-sensitivity analysis yields a frequency shift of less than 1.5% during intensity tuning and an intensity variation of less than 0.8 dB during frequency tuning. The absorber exhibits polarization insensitivity and stable performance under oblique incidence up to 45°. An equivalent circuit model is developed and validated against full-wave simulations. Numerical analyses of fabrication tolerance for the active components confirm that the highly decoupled behavior is robust, with absorption peak shifts below 0.15 GHz and intensity variations below ±1.2 dB. Our conceptual design highlights the potential towards independent multi-parametric control in reconfigurable metasurface absorbers for adaptive electromagnetic shielding, smart radomes, and frequency-agile sensing.

## 1. Introduction

Metasurface absorbers (MAs), as two-dimensional derivatives of metamaterials, have garnered extensive research attention owing to their outstanding capabilities in electromagnetic wave manipulation, subwavelength configuration, and controllable absorption responses [1,2,3,4,5,6]. Over the past decade, diverse MAs have been proposed with single-band [7], dual-band [5], multiband [8], and ultra-wideband [9,10] absorption characteristics, enabling promising applications in radar cross section reduction, electromagnetic shielding, stealth radomes, and wireless communication systems [11,12,13,14]. Nevertheless, conventional passive MAs exhibit fixed electromagnetic responses once fabricated, severely limiting their adaptability in dynamic electromagnetic environments that demand real-time reconfiguration [15,16,17]. To overcome this limitation, intensive efforts have been devoted to developing reconfigurable MAs by introducing active lumped components, phase-change materials, graphene, or liquid metals [14,18,19,20]. Among diverse tuning strategies, the integration of PIN diodes and varactor diodes has become the most prevailing and reliable scheme owing to high compatibility, fast switching speed, low cost, and compatibility with printed circuit board fabrication [21]. In such structures, PIN diodes are generally employed as variable resistors to regulate absorption intensity, while varactors serve as voltage-controlled capacitors to shift absorption frequencies [8,22,23].

Despite substantial advancements, most reported reconfigurable MAs suffer from inevitable cross-coupling between intensity and frequency tuning, meaning that adjustment of one parameter inevitably disturbs the other, which significantly degrades the independence and stability of dynamic regulation [24,25]. Although recent works have attempted independent multiband control [26,27], a critical examination reveals that the degree of decoupling remains largely qualitative. Residual frequency shifts of approximately 5% during intensity tuning [26] and intensity variations exceeding 2 dB during frequency tuning [27] are commonly observed. In addition, most designs rely on complex multilayer configurations or external RF choke elements for bias isolation, which increases fabrication complexity and may introduce additional parasitic effects [20,28,29]. Therefore, the development of a dual-band independently reconfigurable MA with simple configuration, stable operation, polarization insensitivity, and wide-angle stability remains a critical challenge for high-performance intelligent EM devices.

Notably, our group has previously demonstrated an experimentally validated flexible ultra-wideband reconfigurable absorber using PIN diodes integrated in a comparable multilayer configuration with a simplified peripheral bias network [7]. The close agreement between measured and simulated results across 2.5–12 GHz confirmed the reliability of the adopted full-wave simulation methodology and the practicality of the choke-free biasing strategy, providing a solid foundation for the present study. Therefore, the development of a dual-band independently reconfigurable MA that achieves quantitatively verified decoupled control, features a simplified bias network, and maintains polarization insensitivity with wide-angle stability remains a critical challenge.

In this work, we propose a dual-band reconfigurable metasurface absorber with numerically demonstrated control of absorption intensity and absorption frequency. The novelty of the proposed design lies not in the individual use of PIN diodes or varactors, but in the spatially separated integration of these two active elements into different resonant branches. The PIN-diode-loaded branch mainly regulates the low-frequency absorption intensity, whereas the varactor-loaded branch mainly tunes the high-frequency absorption frequency. Full-wave simulations, cross-sensitivity analysis, and equivalent circuit modeling are used to evaluate and interpret the proposed tuning mechanism. A high-absorption band spanning 4.1–7.7 GHz (S_11_ < −10 dB) is obtained, where the absorption intensity can be flexibly regulated via the PIN diode bias without frequency shifting. Simultaneously, a continuously tunable absorption band from 13.4 to 14.4 GHz is achieved by varying the varactor bias, with negligible mutual interference between the two tuning mechanisms. The proposed absorber exhibits favorable polarization insensitivity and stable performance. An equivalent circuit model is developed and independently reproduces the decoupled tuning behavior. The presented conceptual design offers a potential approach for independent multifunctional control in reconfigurable metasurface absorbers, with strong potential for adaptive electromagnetic shielding, smart radomes, and frequency-agile sensors.

## 2. Structure Design and Results Analysis

### 2.1. Structure Design of the MA

**Figure 1:**
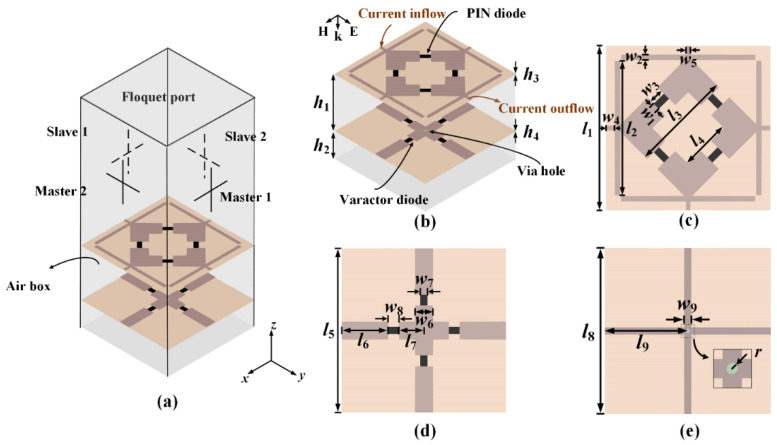
Schematic diagrams of the MA structure: (**a**) 3D simulation model. (**b**) Unit cell structure. (**c**) Top view of the top layer. (**d**) Top view of the middle layer front side. (**e**) Top view of the middle layer back side.

The three-dimensional simulation model of the proposed reconfigurable metamaterial absorber is illustrated in Figure 1a,b. The structure is developed based on a modified sandwich configuration. A typical metamaterial absorber consists of a three-layer stack: an impedance-matched metasurface layer, a dielectric substrate, an air spacer, and a metallic ground plane. In this work, an improved hierarchical design is introduced by inserting an additional metallic-patterned dielectric layer within the air spacer, forming a double-layer metasurface absorber as depicted in Figure 1b.

Both the top and middle metallic patterns are fabricated using copper with an electrical conductivity of *σ* = 5.8 × 10^7^ S/m. The detailed geometry of the top-layer pattern is depicted in Figure 1c. It consists of four symmetric V-shaped metallic strips with open gaps, arranged in a fourfold rotational symmetric manner along the x- and y-axes to achieve broadband absorption. PIN diodes are symmetrically loaded at the gaps of the top-layer resonators. A distinct advantage of this configuration is that bias lines routed along one axis can feed all PIN diodes simultaneously, greatly simplifying the biasing network. The bias traces are narrow and are printed peripherally around the metallic resonators, deliberately placed away from the main resonant current paths. These narrow traces are routed peripherally around the metallic resonators rather than across the regions with a strong resonant current. Therefore, their direct scattering and capacitive loading on the main resonant paths are reduced. In addition, the narrow and elongated bias traces behave as high-impedance DC feeding paths at microwave frequencies, which helps suppress RF leakage toward the external DC supply.

The middle-layer pattern adopts a cross-shaped resonator, with varactor diodes loaded at its central gaps. The bias lines for the varactors are routed on the backside of the middle dielectric substrate and connected to the varactors through metallized via located exactly at the center of the cross-shaped copper pattern. This central position coincides with a virtual RF ground of the cross resonator under symmetrical excitation, further minimizing RF loading. Combined with the physical separation provided by the dielectric substrate, this layout effectively isolates the DC biasing network from the RF fields on the front side.

The dielectric substrate is polyimide (PI) with a relative permittivity *ε_r_* = 3.5 and loss tangent tan *δ* = 0.008. Full-wave modeling and simulation are carried out using ANSYS High-Frequency Structure Simulator (HFSS) 2023. An air box is placed above the multilayer structure, with a Floquet port excitation applied at the top to simulate normally incident plane waves. Master/slave boundary conditions are assigned to the lateral sides to emulate an infinite periodic array.

In the full-wave simulation, the PIN diodes and varactors are modeled using lumped RLC boundary conditions. For the purpose of demonstrating the fundamental decoupling mechanism, the PIN diodes in forward bias are represented as ideal variable resistors, and the varactors in reverse bias as ideal variable capacitors, with package parasitic reactances initially neglected. It should be noted that typical commercial surface-mount PIN diodes (Skyworks SMP1340-079LF) (Skyworks Solutions, Inc., Woburn, MA, USA) exhibit series inductance below 1 nH and package capacitance below 0.3 pF, while hyperabrupt varactors (MACOM MAVR-000120-1411) (MACOM Technology Solutions Inc., Lowell, MA, USA) offer a capacitance tuning range from about 0.15 pF to over 0.7 pF. These realistic parasitics are small enough that they do not alter the fundamental decoupling behavior demonstrated here.

### 2.2. Performance Analysis of the MA

Since the metasurface has a sub-wavelength periodic structure, its performance can be analyzed by means of effective medium theory. The absorption rate (AR) is defined as:

(1) $$AR=1-RR-TR=1-{\left|S_{11}\right|}^{2}-{\left|S_{21}\right|}^{2}$$

where *RR* and *TR* are reflection and transmission, and *S*_11_ and *S*_21_ are reflection and transmission coefficients, respectively. The absorption performance depends on both the reflection coefficient *S*_11_ and the transmission coefficient *S*_21_. As the metallic backplane prevents transmission, *S*_21_ ≈ 0, and the absorption characteristics can be fully described by *S*_11_.

**Figure 2:**
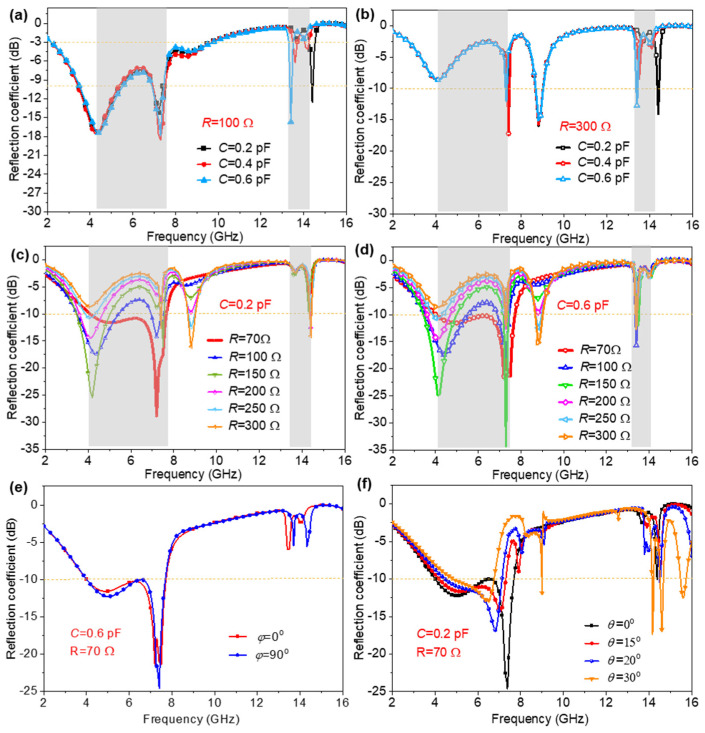
Reflection coefficient S_11_ curves of the MA: (**a**) *R* = 100 Ω; (**b**) *R* = 300 Ω; (**c**) *C* = 0.2 pF; (**d**) *C* = 0.6 pF; (**e**) different polarization angles *φ*; (**f**) different incident angles *θ*.

The simulated reflection coefficient curves are plotted in Figure 2. Here, *R* represents the equivalent resistance of the PIN diodes, and *C* represents the equivalent capacitance of the varactors. The selected tuning ranges are associated with the effective RF-equivalent parameters of the selected commercial diodes. The chosen resistance range (70–300 Ω) corresponds well to the forward dynamic resistance variation in commercial PIN diodes (e.g., SMP1340-079LF) under bias currents from tens of mA down to near-zero. The capacitance range (0.2–0.6 pF) is a practical subset of the reverse-bias tuning curve of hyperabrupt varactors (e.g., SMV1231-079LF), confirming the physical realizability of the parametric study.

Comparing Figure 2a,b, the PIN diode resistance *R* mainly affects the absorption intensity in the low-band region. As *R* increases from 100 Ω to 300 Ω, the absorption intensity varies from −6.55 dB to −1.86 dB in the low-band, while the high-band remains nearly unchanged. By comparing Figure 2c,d, the varactor capacitance *C* mainly shifts the high-band absorption peak from 14.4 GHz to 13.4 GHz as *C* increases from 0.2 pF to 0.6 pF, with little influence on the low-band absorption. For R = 70 Ω, the lower absorption band with S_11_ < −10 dB spans from 4.1 GHz to 7.7 GHz. This demonstrates that varying *C* continuously shifts the absorption frequency while leaving the bandwidth and absorption intensity of the broadband absorption region unaffected.

Quantitative cross-sensitivity analysis shows that when R varies from 70 to 300 Ω at fixed C, the center frequency of the intensity-reconfigurable band shifts by <1.5% and the frequency-tunable peak shifts by <0.5%. When C varies from 0.2 to 0.6 pF at fixed R, the absorption intensity at the tunable peak changes by <0.8 dB, and the broadband |S_11_| remains within 1.2 dB. These values are well below commonly accepted thresholds for significant cross-coupling.

Furthermore, the frequency range where *S*_11_ > −3 dB between the two reconfigurable bands is defined as the isolation band. For *C* = 0.2 pF and 0.6 pF, the relative bandwidths of the isolation band reach 38.6% and 44.2%, respectively. Taking *R* = 70 Ω and *C* = 0.6 pF as a typical case, Figure 2e shows the reflection coefficients under different polarization angles *φ*. The curves remain nearly overlapping, verifying that the absorber is polarization-insensitive. Similarly, Figure 2f illustrates that the absorber maintains robust performance under oblique incidence angles *θ*, demonstrating favorable angular stability. After structural optimization and parametric sweeping, the final geometrical parameters of the MA are summarized in Table 1.

**Figure 3:**
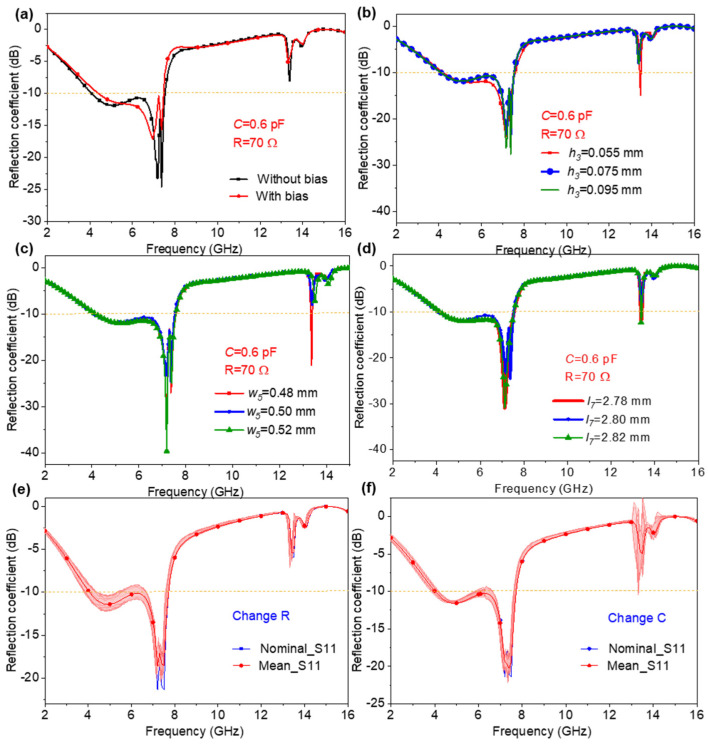
Reflection coefficient S_11_ curves of the MA for simulation-evaluated fabrication tolerance: (**a**) with/without the bias lines. Under ±0.02 mm variation in three critical parameters: (**b**) PI thickness *h*_3_; (**c**) bias line width *w*_5_; and (**d**) resonator length *l*_7_. (**e**) Statistical distribution of S_11_ under ±10% PIN diode resistance variation. (**f**) Statistical distribution of S_11_ under ±10% varactor capacitance variation.

To evaluate the influence of the bias network on the absorber performance, full-wave simulations were performed with the bias lines represented by choke inductors at the feed points of the active devices. Figure 3a compares the model with and without the top-layer bias lines over 2–16 GHz. The overall RMS deviation is only 0.57 dB, with 90.4% of frequency points exhibiting a deviation below 1.0 dB. In the intensity-reconfigurable band (4.1–7.7 GHz), the S_11_ < −10 dB bandwidth is largely preserved, with the resonant peaks shifting by approximately 0.21 GHz and an in-band RMS deviation of 0.65 dB. In the frequency-reconfigurable band (13.4–14.4 GHz), the absorption peak frequency remains essentially unchanged (<0.05 GHz shift). These results confirm that the bias network introduces only a negligible perturbation to the RF performance.

The robustness of the design against manufacturing inaccuracies was evaluated by individually varying three critical geometrical parameters. Full-wave simulations were performed for each perturbed geometry, and the resulting S_11_ curves were compared with the nominal design. In the intensity-reconfigurable band (4.1–7.7 GHz), the maximum in-band |S_11_| deviation remained below 1.1 dB across all trials, and the S_11_ < −10 dB fractional bandwidth changed by less than 5%. In the frequency-reconfigurable band, the absorption peak shifted by less than 0.1 GHz, and the peak intensity varied by less than 1.2 dB. These results confirm adequate resilience against typical fabrication and component tolerances.

In addition to geometrical tolerances, a statistical sensitivity analysis of the active components was performed. Twenty independent full-wave simulations were carried out for each component, with *R* and *C* randomly varied within ±10% of their nominal values (*R*_0_ = 70 Ω, *C*_0_ = 0.6 pF). The results are shown in Figure 3e,f. For PIN diode tolerance, in the intensity-reconfigurable band (4.1–7.7 GHz), the absorption peak frequency shifts by less than 0.1 GHz, and the *S*_11_ value at the peak varies within ±1.0 dB (standard deviation < 0.35 dB). The influence on the high-band (13–15 GHz) is negligible, with *S*_11_ variations below 0.3 dB. For varactor tolerance, in the frequency-reconfigurable band (13.4–14.4 GHz), the absorption peak frequency shifts by less than 0.15 GHz, and the peak intensity varies within ±1.2 dB. The low-band (4–8 GHz) *S*_11_ remains almost unchanged. Even under worst-case simultaneous tolerances, the frequency shift during intensity tuning stays below 0.2 GHz, and the intensity variation during frequency tuning stays below 1.3 dB. These results confirm that the proposed design is robust against realistic component variations.

### 2.3. Absorption Mechanism Analysis

**Figure 4:**
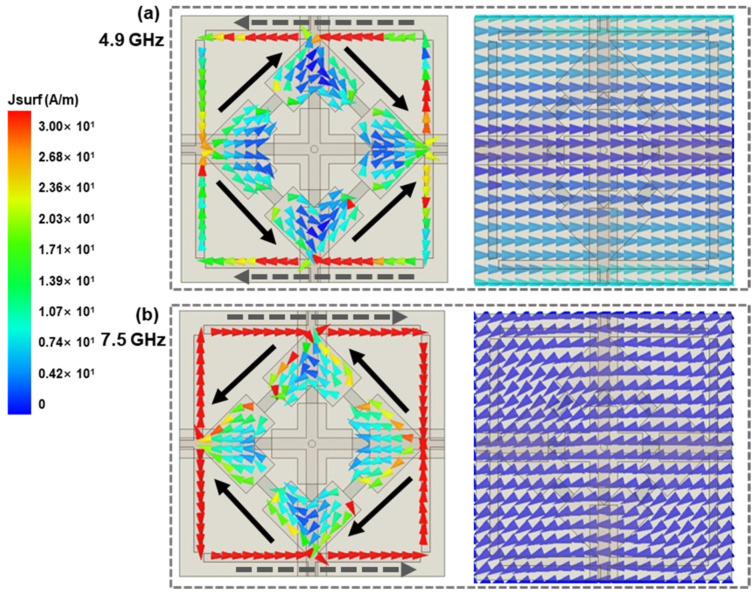
Surface current distributions of the top metallic pattern and metallic ground plane of the MA: (**a**) 4.9 GHz; (**b**) 7.5 GHz.

To further clarify the absorption mechanism of the proposed MA, the surface current distributions are analyzed in detail. For *C* = 0.2 pF and *R* = 70 Ω, two distinct resonant peaks are observed in the intensity-reconfigurable band at *f* = 4.9 GHz and 7.5 GHz. Figure 4 depicts the surface current distributions on the top metallic pattern and the ground plane at these resonant frequencies. At the lower-band resonances (Figure 4), the currents are predominantly concentrated on the central V-shaped patches, creating a resonant mode strongly influenced by the resistance of the PIN diodes loaded at their gaps. At *f* = 4.9 GHz, the surface currents on the central V-shaped metallic patches flow in the same rightward direction as those induced on the ground plane, while the currents on the peripheral metallic branches circulate in an antiparallel direction relative to the ground plane. At *f* = 7.5 GHz, the currents on the V-shaped patches and the ground plane flow leftward, and the peripheral metallic branches still exhibit antiparallel current directions with respect to the ground plane. Such a configuration excites both electric and magnetic resonances simultaneously.

**Figure 5:**
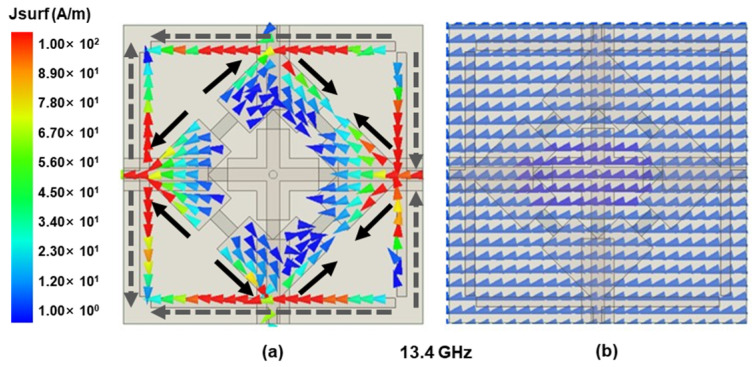
Surface current distributions of the MA at *f* = 13.4 GHz: (**a**) top metallic pattern; (**b**) metallic ground plane.

For C = 0.6 pF and R = 70 Ω, the absorption peak in the frequency-reconfigurable region is located at *f* = 13.4 GHz. Figure 5 presents the surface current distributions at this frequency. In contrast to the lower band, the upper-band current distribution (Figure 5) spreads more uniformly across the entire cross-shaped resonator, explaining why this mode is preferentially sensitive to the varactor capacitance placed at the central gaps. The currents on the left and right V-shaped patches, as well as the peripheral metallic branches, flow in the same direction as the ground plane currents, whereas the currents on the top and bottom V-shaped patches flow antiparallel to the ground plane currents. This combined current distribution further verifies the co-excitation of electric and magnetic resonances.

The quality factors (Q-factors) of the resonances, extracted from the −3 dB bandwidths of the absorption peaks, are approximately 12 at 4.9 GHz and 45 at 13.4 GHz, indicating moderate selectivity in the lower intensity-reconfigurable band and high selectivity in the upper frequency-reconfigurable band, respectively. The corresponding absorption efficiencies at resonance both exceed 90%. Furthermore, according to the simulated current distribution, this region exhibits a relatively weak RF perturbation for the dominant resonant mode. Therefore, the backside routing and symmetry-point via connection help reduce the interaction between the bias network and the RF resonant currents.

**Figure 6:**
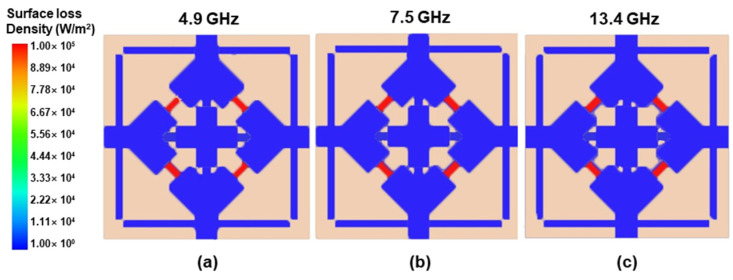
Power loss density distributions on the surface of the MA: (**a**) 4.9 GHz; (**b**) 7.5 GHz; (**c**) 13.4 GHz.

In both passbands, efficient electromagnetic absorption originates from the strong coupling and synergistic effect between the top metallic pattern and the metallic ground plane, where the synergistic electromagnetic resonances provide the dominant energy dissipation for the proposed structure. Figure 6 displays the power loss density distributions at the corresponding resonant frequencies of 4.9 GHz, 7.5 GHz, and 13.4 GHz. For both the intensity-reconfigurable and frequency-reconfigurable regions, the power loss density is highly concentrated at the positions of the lumped PIN diodes, indicating that the absorption mechanism is dominated by Ohmic losses.

## 3. Equivalent Circuit Analysis

**Figure 7:**
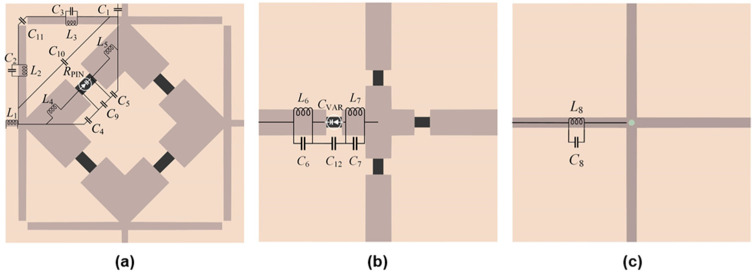
(**a**) Correspondence between the top layer and the equivalent circuit. (**b**) Correspondence between the middle layer and the equivalent circuit. (**c**) Correspondence between the middle layer feed lines and the equivalent circuit.

To validate the independent reconfigurability of the proposed MA, an equivalent circuit model (ECM) is established and employed for theoretical analysis and simulation verification. Based on the “trace inductance” and “gap capacitance” correspondence rules, the ECM is constructed as illustrated in Figure 7. Benefiting from the fourfold rotational symmetry of the metallic pattern, only one quadrant of the unit cell needs to be considered in the equivalent circuit derivation, which simplifies the modeling process significantly via series-parallel combination rules. In this model, the PIN diodes are represented by a variable resistor *R_PIN_* to characterize their bias-dependent resistance, while the varactor diodes are modeled as a variable capacitor *C_VAR_* to reflect their voltage-tunable capacitance.

**Figure 8:**
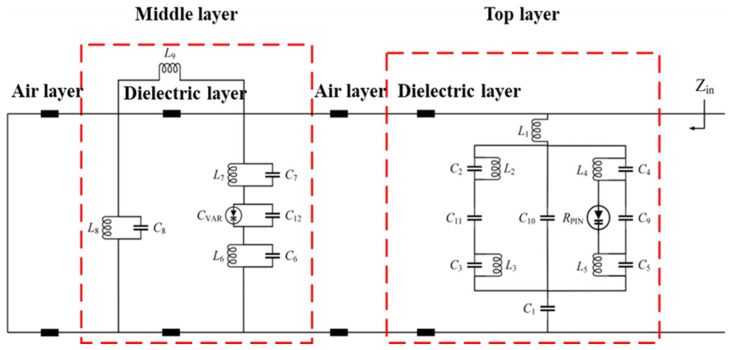
Equivalent circuit of the MA.

The complete equivalent circuit of the absorber is depicted in Figure 8, where the top and middle metallic layers are electromagnetically cascaded to form a multilayer impedance-matching system. The metallic ground plane beneath the air spacer of the middle layer is modeled as a short-circuited transmission line, which perfectly blocks electromagnetic wave transmission and reflects all incident energy to the upper layers. Since a metallized via interconnects the front and back metallic patterns at the center of the middle dielectric substrate, a small inductor is incorporated in parallel with the transmission line segment representing the middle substrate to account for the parasitic inductance introduced by the via, ensuring the accuracy of the circuit model at high frequencies.

The Advanced Design System (ADS) software (2025 Update 2.3) is employed to optimize the lumped inductance and capacitance values in the equivalent circuit model. Because of the multiple coupled electromagnetic resonances between the stacked layers, a direct analytical extraction of all parasitic elements is impractical. Therefore, the lumped values were obtained through numerical optimization in ADS to match the full-wave S-parameters. The physical plausibility of the optimized values was verified by order-of-magnitude estimates based on the metallic pattern geometry and standard microstrip formulas. The fact that the ECM, using a completely different computational framework, independently reproduces the highly decoupled tuning behavior provides robust cross-validation of the design.

Due to the central symmetry of the metallic pattern, the components *C*_2_ and *C*_3_, *L*_2_ and *L*_3_, *C*_4_ and *C*_5_, *L*_4_ and *L*_5_ are assigned identical values to maintain the symmetry of the circuit. The optimized lumped component parameters are summarized in Table 2, which are used to simulate the reflection coefficient of the absorber and compare with the full-wave electromagnetic simulation results obtained from ANSYS HFSS.

**Figure 9:**
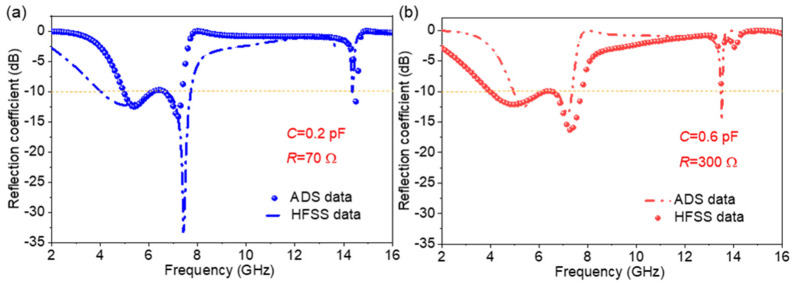
Comparison of reflection coefficient simulation results between HFSS and ADS for the MA under (**a**) *C* = 0.2 pF, *R* = 70 Ω, and (**b**) *C* = 0.6 pF, *R* = 300 Ω, respectively.

The reflection coefficient *S*_11_ obtained from the equivalent circuit simulation is compared with the HFSS full-wave simulation results in Figure 9. Figure 9a and Figure 9b show the comparison for *R* = 70 Ω and *R* = 300 Ω, respectively, under different varactor capacitance values. The comparison results clearly validate that adjusting the equivalent resistance of the PIN diodes only modulates the absorption intensity in the intensity-reconfigurable passband, while tuning the equivalent capacitance of the varactor diodes solely shifts the absorption frequency in the frequency-reconfigurable passband. The equivalent circuit model accurately reproduces the highly independent reconfigurability of absorption intensity and frequency observed in full-wave electromagnetic simulations, which strongly verifies the feasibility and validity of the proposed MA design.

A minor discrepancy in the bandwidth of the intensity-reconfigurable region is observed between HFSS and ADS simulation results. This discrepancy arises because the simplified cascaded single-resonance branches of the ECM cannot fully capture the broadband near-field interlayer coupling between the stacked top and middle metallic layers, which is inherently a limitation of the lumped-element approach for representing complex distributed electromagnetic interactions.

To highlight the advances of the proposed design, Table 3 provides a systematic comparison with representative reconfigurable metasurface absorbers from the recent literature. The evaluation covers tuning mechanisms, frequency bands, degree of independent control, quantitative cross-coupling metrics, bias network complexity, and angular/polarization stability.

As summarized in Table 3, most existing designs provide only single-function tunability or operate in a single band. Even in structures that combine PIN and varactor diodes, the tuning mechanisms are typically coupled: Ref. [26] exhibits a residual frequency shift of approximately 5% during intensity tuning, and Ref. [27] shows intensity variations greater than 2 dB during frequency tuning. The proposed absorber achieves highly decoupled dual-band independent reconfiguration with cross-coupling metrics below 1.5% in frequency and 0.8 dB in intensity. The simplified bias network, polarization-insensitive wide-angle performance, and verified fabrication tolerance further distinguish this work.

## 4. Conclusions

In this work, a dual-band reconfigurable metasurface absorber incorporated with lumped active components is proposed. By integrating PIN diodes and varactor diodes into the top and middle metallic layers respectively, numerically demonstrated highly decoupled control of absorption intensity and frequency is realized without obvious cross-coupling. Quantitative cross-sensitivity metrics confirm that the two tuning mechanisms operate with negligible mutual interference—frequency shifts remain below 1.5% during intensity tuning, and intensity variations remain below 0.8 dB during frequency tuning. An intensity-reconfigurable band (4.1–7.7 GHz) and a frequency-reconfigurable band (13.4–14.4 GHz) are realized, with polarization insensitivity and angular stability up to 45°. Surface current analysis reveals that the decoupled operation originates from distinct spatial distributions of the resonant modes. An equivalent circuit model independently reproduces the tuning behavior, while bias network analysis confirms minimal RF perturbation (RMS deviation 0.57 dB). Numerical analyses of fabrication tolerance further verify the design robustness against manufacturing variations. Thus, this work serves as a numerical proof-of-concept for decoupled dual-band reconfigurable absorbers, offering a clear design guideline and quantitative performance benchmarks for subsequent experimental efforts.

## Figures and Tables

**Table 1:** The structural parameters of the MA.

Parameter	Value (mm)	Parameter	Value (mm)	Parameter	Value (mm)
*h*_1_	6	*l*_5_	18.385	*w*_4_	1
*h*_2_	5	*l*_6_	5.192	*w*_5_	0.5
*h*_3_	0.075	*l*_7_	2.8	*w*_6_	2
*h*_4_	0.025	*l*_8_	18.385	*w*_7_	0.8
*l*_1_	18.385	*l*_9_	9.192	*w*_8_	1.2
*l_2_*	15	*w*_1_	0.8	*w*_9_	0.8
*l*_3_	11.314	*w*_2_	0.6	*r*	0.25
*l*_4_	5.657	*w*_3_	1.6		

**Table 2:** The parameters of the equivalent circuit for the MA.

Parameter	Value	Parameter	Value	Parameter	Value
*L*_1_	2.3077 nH	*L*_8_	26 nH	*C*_6_	14.0086 pF
*L*_2_	80 nH	*L*_9_	2.8 nH	*C*_7_	0.15 pF
*L*_3_	80 nH	*C*_1_	0.12 pF	*C*_8_	20 pF
*L_4_*	0.208 nH	*C*_2_	60 pF	*C*_9_	0.01 pF
*L*_5_	0.208 nH	*C*_3_	60 pF	*C*_10_	0.1288 pF
*L*_6_	100 nH	*C*_4_	1.9 pF	*C*_11_	0.1 pF
*L*_7_	50 nH	*C*_5_	1.9 pF	*C*_12_	0.1 pF

**Table 3:** Comparison of the proposed absorber with representative reconfigurable metasurface absorbers reported in the literature.

Ref.	Active Element(s)	Frequency Bands (GHz)	Independent Tuning	Cross-Coupling/Decoupling Evidence	Independent Tuning Capability	Angular Stability & Polarization
[2]	Varactors (freq. tuning)	Single narrowband (C-band)	No	Not mentioned	Simple	Not specified
[7]	PIN diodes (intensity)	5.4–16.77 (ultra-wideband)	No (intensity only)	Not mentioned	Moderate; peripheral traces, no lumped chokes	Polarization-sensitive, up to 30°
[8]	PIN + varactors (shared layer)	6–14 (coupled)	No (functions combined)	Frequency shift observed during mode switching	Complex; shared bias	Up to 30°, polarization-dependent
[20]	PIN diodes (switching)	3.5–9.5 (absorber/reflector)	No (binary switching only)	Not mentioned	Multilayer FSS, high profile	Up to 30°, polarization-sensitive
[23]	PIN diodes (binary states)	X-band	No (two-state switching)	Not mentioned	Simple	Up to 20°
[26]	PIN + varactors	3–6 (intensity) + 8–12 (freq.)	Partial	Δ*f* ≈ 5% when R varied	Requires RF choke inductors	Up to 30°
[27]	PIN + varactor (shared layer)	6.5–11.2 (single band)	No (single band, coupled)	Δ*S*_11_ > 2 dB during freq. tuning	Multilayer, decoupling circuit	Up to 40°, polarization-insensitive
This work	PIN diodes + varactors	Band I: 4.1–7.7 (intensity-rec.) Band II: 13.4–14.4 (freq.-rec.)	Yes, highly decoupled	Δ*f* < 1.5%, Δ*S*_11_ < 0.8 dB in cross-tuning	Simplified; peripheral traces (0.5 mm)	Up to 45°, polarization-insensitive

## Data Availability

The original contributions presented in this study are included in the article. Further inquiries can be directed to the corresponding authors.
